# Opsin gene expression regulated by testosterone level in a sexually dimorphic lizard

**DOI:** 10.1038/s41598-018-34284-z

**Published:** 2018-10-30

**Authors:** Wen-Hsuan Tseng, Jhan-Wei Lin, Chen-Han Lou, Ko-Huan Lee, Leang-Shin Wu, Tzi-Yuan Wang, Feng-Yu Wang, Duncan J. Irschick, Si-Min Lin

**Affiliations:** 1 Department of Life Science, National Taiwan Normal University, Taipei, 116 Taiwan; 2 Department of Animal Science and Technology, National Taiwan University, Taipei, 106 Taiwan; 3 Biodiversity Research Center, Academia Sinica, Taipei, 115 Taiwan; 4 National Applied Research Laboratories, Taiwan Ocean Research Institute, Kaohsiung, 801 Taiwan; 5 Department of Biology, 221 Morrill Science Center, University of Massachusetts, Amherst, MA 01003 USA

## Abstract

Expression of nuptial color is usually energetically costly, and is therefore regarded as an ‘honest signal’ to reflect mate quality. In order to choose a mate with high quality, both sexes may benefit from the ability to precisely evaluate their mates through optimizing visual systems which is in turn partially regulated by opsin gene modification. However, how terrestrial vertebrates regulate their color vision sensitivity is poorly studied. The green-spotted grass lizard *Takydromus viridipunctatus* is a sexually dimorphic lizard in which males exhibit prominent green lateral colors in the breeding season. In order to clarify relationships among male coloration, female preference, and chromatic visual sensitivity, we conducted testosterone manipulation with mate choice experiments, and evaluated the change of opsin gene expression from different testosterone treatments and different seasons. The results indicated that males with testosterone supplementation showed a significant increase in nuptial color coverage, and were preferred by females in mate choice experiments. By using quantitative PCR (qPCR), we also found that higher levels of testosterone may lead to an increase in rhodopsin-like 2 (*rh2*) and a decrease in long-wavelength sensitive (*lws*) gene expression in males, a pattern which was also observed in wild males undergoing maturation as they approached the breeding season. In contrast, females showed the opposite pattern, with increased *lws* and decreased *rh2* expression in the breeding season. We suggest this alteration may facilitate the ability of male lizards to more effectively evaluate color cues, and also may provide females with the ability to more effectively evaluate the brightness of potential mates. Our findings suggest that both sexes of this chromatically dimorphic lizard regulate their opsin expression seasonally, which might play an important role in the evolution of nuptial coloration.

## Introduction

The amazing variety of sexual coloration in sexually dimorphic animals is one of the most intriguing phenomena for evolutionary biologists, as noted by Charles Darwin when he first described sexual selection. Evolution of the sensory system and behavior are often correlated with sexual coloration in many different animals, and this process may also affect how speciation occurs^1–6^. In mating systems with strong sexual selection, the efficacy of signals is often critical for both signal transmitter and receiver^7,8^. Indeed, even subtle differences in visual sensitivity could influence how individuals within the same species interact with one another^9–12^.

Theoretically, sexual traits in males will evolve in concert with female preference, which is highly dependent on precision of signal recognition^13^. In some animals, the expression of these sexual traits is regulated in part through the expression of testosterone^14–16^. In many male vertebrates, the androgenic sex steroid testosterone is directly associated with reproductive investment through enhancement of the expression of secondary sexual characters. Further, circulating levels of testosterone have also been shown to influence home range size, activity, mobility, aggressiveness, and sexual behavior, which in some cases can influence mating success^16–20^.

Along with effective transmission of signals is the need for a precise evaluation of courtship coloration expression. This coordination is critical for signal receivers both in females (for choosing a high-quality mate) and in males (for assessing the quality of potential opponents). A key part of this coordination is spectral sensitivity. Spectral sensitivity relies on the presence of photoreceptor cells in animal retina. Opsins contribute the major function in these cells; they are proteins composed of about 350 amino acids that are folded into a seven transmembrane α-helices structure, forming a binding pocket encompassing the retinal (chromophore). Rod cells contain rhodopsin (Rh1), which is responsible for dim light vision (scotopic), while cone cells are responsible for bright-light and chromatic vision (photopic). Except for diurnal geckos, which possess only three pigments (lack of short wavelength sensitive 2, SWS2)^21,22^, tetrachromatic vision is widely found in diurnal lizards^23–25^. Their cone cells could be divided into four groups which are sensitive to different wavelengths: UV-, short-, middle- and long-wavelength-sensitive photoreceptors, which comprise short wavelength sensitive 1 (SWS1), SWS2, rhodopsin like (Rh2) and long wavelength sensitive (LWS) opsins, respectively^26,27^.

Several mechanisms have been proposed to explain how animals have evolved such structures for particular spectra. The first route is to modify the classes and numbers of opsins from the ancestral state, which is usually observed in organisms that share a nocturnal, deep sea, and fossorial lifestyle^28–32^. The second route is amino acid substitution in opsins which alters the affinity to associated chromophore and causes the shift of maximum absorption wavelength (λ_max_)^33–35^. Given restricted genetic divergence, intraspecific difference in spectral sensitivity is not likely to originate from the previous two mechanisms, but is more likely to be generated by some other routes, such as alternative splicing of visual opsins^36^, or upstream regulation in opsin genes^37^. Variation in the regulation of opsin genes has been reported among individuals within species^13,38^ or within individual levels^39–42^. However, the majority of these studies focused on specializations to surrounding photic environments, and have not investigated seasonal alteration of nuptial coloration and mate choice behavior. Three-spined sticklebacks (*Gasterosteus aculeatus*) are one of the few cases in which such factors (including body condition) was considered^43^. Except for a few cases^44^, prior work has not examined the role of opsin gene expression and sexual selection in terrestrial vertebrates.

Some sexually dimorphic species within the lizard family Lacertidae provide an opportunity to investigate this issue. The East Asian *Takydromus* lizards, comprising about 20 species, present substantial variation in their courtship systems, even among closely related species^45,46^. *Takydromus viridipunctatus* might be the most well-studied species in this genus, and is commonly found in regions of northern Taiwan^47^. In grasslands of early succession stage, they sometimes form huge population size in high density.

**Figure 1:**
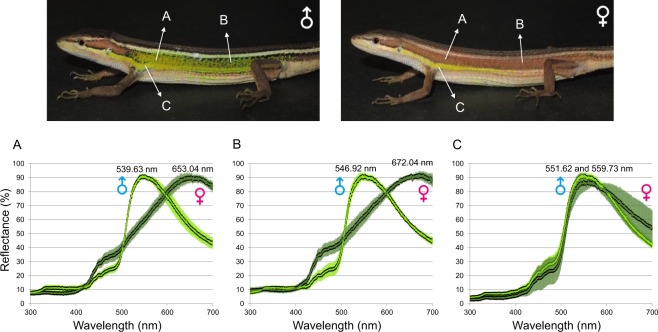
Nuptial coloration measured and plotted as percent reflectance spectra (mean values with standard deviation) from 6 males and 6 females in the breeding season. The white arrows indicate color patches which we measured from (**A**) anterior lateral regions; (**B**) posterior lateral regions; and (**C**) ventrolateral green lines. Differences in reflectance between two sexes are found at their lateral side.

During the breeding season, male lizards present bright green spots on their lateral sides, which might play a role both for mate recognition and/or signaling male quality (Fig. 1). At the same time, some females also show a lateral green line that is more lightly colored. While the function of this stripe remains unclear, it also could be employed as a signal during the mating process. The exhibition of nuptial color in males of this species has recently been shown to impose an energetic cost, as there is a significant correlation among males between color expression and both ectoparasite loads and mortality (Lin *et al*., unpublished data; and also see Shaner *et al*., 2013 for discussion on ectoparasite loads). Therefore, augmentation of opsin gene expression during the breeding season might provide a route to optimize the visual sensitivity of breeding adults.

In this study, we conducted a series of behavioral and molecular experiments to examine links among sexual hormones, seasonality, sexual maturity, nuptial color, mate choice, and opsin gene expression among individuals of the lizard *Takydromus viridipunctatus*. First, we hypothesized that testosterone (abbreviated as T in the experimental treatments) plays a crucial rule in sexual selection, which could be justified by the enhancement of male nuptial coloration and female preference after testosterone treatments. Second, we hypothesized that both sexes of this lizard may adjust their color visual sensitivity via regulation of opsin gene expression.

## Results

### Nuptial color reflectance

Spectra from the nuptial color of the grass lizard are shown in Fig. 1. The lateral green spots of the males reflect middle to long wavelength (anterior lateral green spots: peak wavelength 539.63 nm; reflectance 89.7 ± 2.3%; posterior lateral green spots: peak wavelength 546.92 nm, reflectance 91.5 ± 3.0%), displaying shiny green color (Fig. 1A,B). In contrast, females differ notably from males by reflecting brownish color on their lateral sides (anterior lateral brown belt: peak wavelength 653.04 nm; reflectance 90.5 ± 3.0%; posterior lateral green spots: peak wavelength 672.04 nm, reflectance 92.1 ± 3.2%). Nevertheless, mature females are sometimes capable of representing a ventrolateral green line (peak wavelength 559.73 nm, reflectance 87.3 ± 4.3%), which represents similar reflectance spectra with those from males (peak wavelength 551.62 nm, reflectance 91.7 ± 2.7%; Fig. 1C).

### Nuptial color coverage enhanced by testosterone treatment

Testosterone levels from feces of male lizards from the behavior experiments were significantly different among treatments (Kruskal-Wallis test: *χ*^2^ = 24.46, p < 0.0001). Mean testosterone levels of males from the high testosterone (high T) group (221.01 ± 21.22 ng/g) were approximately three times higher than in the control group (69.80 ± 20.68 ng/g); whereas testosterone levels of the medium testosterone (medium T) group was between these two treatments (122.02 ± 20.19). These results showed that the testosterone manipulation was effective, and the T concentration of treated individuals was within 130% of the magnitude of the highest individuals from the wild (173.22 ng/g).

**Figure 2:**
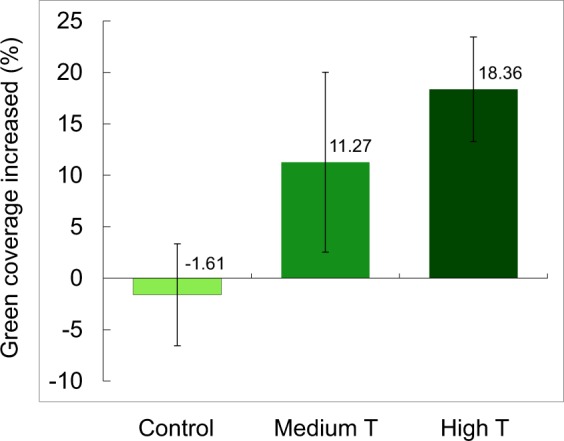
Changes of lateral green color coverage after testosterone (T) treatments on males. The enhancement ratios significantly differed among treatments (Kruskal-Wallis tests: *χ*^2^ = 7.09, df = 2, p = 0.03). Provision of exogenous testosterone significantly enhanced the coverage of the greenish nuptial color (middle and right).

The degree of green coloration before and after testosterone (T) manipulation significantly differed among treatments (Kruskal-Wallis tests: *χ*^2^ = 7.09, df = 2, p = 0.03, Fig. 2). The coverage of the green area increased in high T (18.36%) and medium T (11.27%) groups after one-month testosterone manipulation, but not in control lizards (−1.61%). Provision of testosterone effectively increased the coverage of green color on the lateral side of males.

Although testosterone treatments successfully enhanced the coverage area of nuptial spots, the quality of color did not vary among treatments. The hue values of greenish composition among high-testosterone, medium testosterone, and control groups did not differ statistically before treatments (F_2,57_ = 0.9185, p = 0.4049), or after treatments (F_2,57_ = 0.9746, p = 0.3835). Therefore, we conclude that the effect of testosterone changed the coverage of greenish coloration, but not the quality of the color.

### Female preference on males with high green coverage

**Figure 3:**
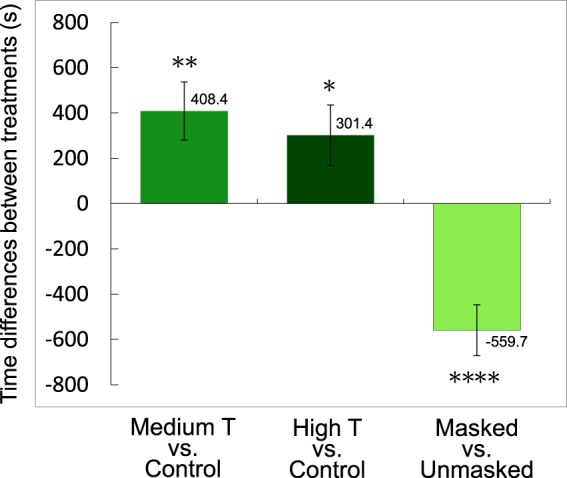
Net time differences (in seconds) between the times spent by a female positioned nearby the two males with different green color coverages within the 1-hour record interval. Compared to controls, medium and high testosterone (T) treated males received a significantly longer attraction from the females for a mean of 408.4 and 301.4 seconds, respectively (left and middle). On the other hand, males masked with pale brown paints lost to the control males for a mean of 559.7 seconds (right). Asterisks denote the statistical significance in pair t-test (*p < 0.05; **p < 0.01; ****p < 0.0001).

Females showed significant preference for males with a large degree of green coverage from T treatment. Among the 26 trials of medium T vs. control pairings, 21 females spent more time close to the T-treated (more greenish) males than the control (pair t-test: t = 3.18, df = 25, p = 0.0039; Fig. 3). A similar situation was found from the high T vs. control pairings: females preferred to spend more time near the more greenish males with high T treatment (18 of the 26 trials; pair t-test: t = 2.26, df = 25, p = 0.03; Fig. 3).

When the masked males (pale brown) are paired with the unmasked ones, females preferred to spend more time nearby the unmasked (green-spotted) males. Negative values (time spent on the masked male minus the unmasked male) were obtained from 19 among the 21 trials, with the time differences being significantly lower than zero (pair t-test: t = 5.00, df = 20, *p* < 0.0001, Fig. 3). These two experiments showed that the lateral green color of male *T. viridipunctatus* could be a preferred visual signal for female choice, and possibly serves as the hint for male quality in the breeding season.

### Alteration in opsin expression

**Figure 4:**
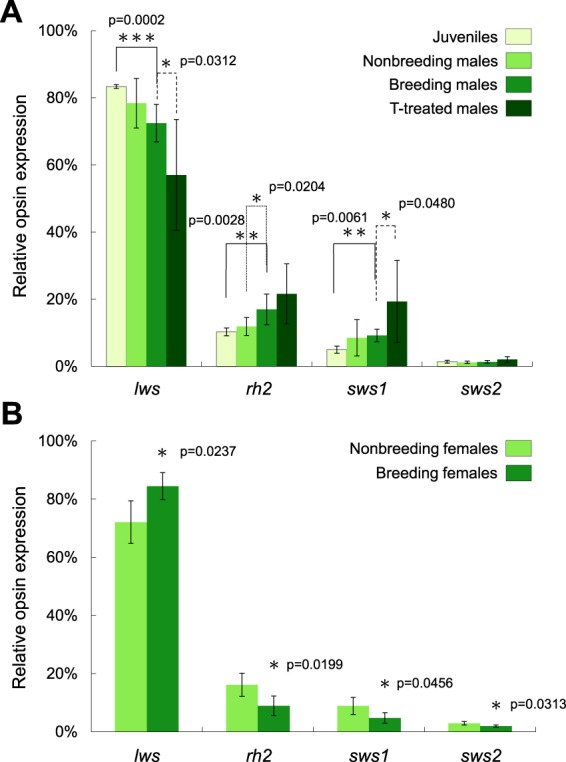
Cone opsin gene expression profiles from (**A**) males and (**B**) females in different seasons and the effect of testosterone treatment. The males showed a prominent trend of increased *rh2* and decreased *lws* expression as approaching the breeding season; while the females showed a reverse pattern. Colored bars shown as mean ± 1 S.D., asterisks mark the statistically significant differences in student’s t-test (*p < 0.05; **p < 0.01; ***p < 0.001) for each cone opsin gene.

Increased expression of *rh2* and *sws1* and decreased expression of *lws* was observed when juvenile males matured (*lws*: t = 3.11, df = 11, *p* = 0.0099; *rh2*: t = 2.31, df = 11, *p* = 0.0414; *sws1*: t = 3.48, df = 11, *p* = 0.0052; Fig. 4A). A similar trend was observed between males in the breeding versus nonbreeding season (significance: *rh2*: t = 2.32, df = 14, *p* = 0.0361). Manipulation of testosterone could further strengthen this trend, as the males with T treatment represented the highest *rh2* and lowest *lws* (Fig. 4A). Statistically, they represented significant increase of *sws1* (t = 2.77, df = 9.41, *p* = 0.0210) and decrease of *lws* (t = 2.71, df = 10.12, *p* = 0.0218) compared to the controls. Another impact of testosterone on males was the significant increase of variation in expression levels (Lavene test, *lws*: *p* < 0.0001; *rh2*: *p* = 0.045; *sws1*: *p* = 0.0003; *sws2*: *p* = 0.098).

In contrast, females showed a completely reversed pattern (Fig. 4B). Relative *lws* expressions were significantly higher in females from the breeding season than non-breeding season (t = 3.08, df = 9, *p* = 0.0132). For the other three classes of cone opsin genes (*rh2*, *sws1*, and *sws2*), expression levels were significantly lower in the breeding season (*rh2*: t = 2.96, df = 9, *p* = 0.0159; *sws1*: Z = 2.28, *p* = 0.0225; *sws2*: t = 2.79, df = 9, *p* = 0.0210; Fig. 4B).

**Figure 5:**
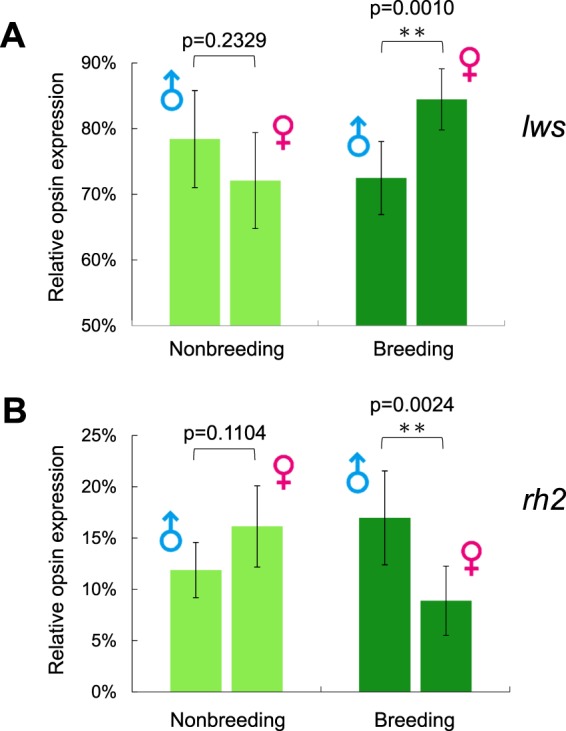
Intersexual comparison of (**A**) *lws* and (**B**) *rh2* between males and females in breeding and non-breeding seasons. Colored bar shown as mean ± 1 S.D.; asterisks mark the statistically significant differences in student’s t-test (**p < 0.01; ***p < 0.001) between the two sexes.

When comparing between males and females collected at the same moment, all opsin genes except for SWS2 (possibly due to the considerably lower expression level) showed significant differences in the breeding season. Breeding females showed higher *lws* expression than males (t = 4.14, df = 14, *p* = 0.0010, Fig. 5A), while breeding males had higher *rh2* expression than females (t = 3.51, df = 14,*p* = 0.0034, Fig. 5B). It is notable that expression difference occurs only in the breeding season. In nonbreeding season, males and females represent a reverse pattern, but with no statistical significance.

Both males and females represent changes in the expression level of opsin genes in the breeding season. Males show high expression in *rh2* and low expression in *lws*; while females show the reverse. Sexual maturity and T treatment led to the same effect on males as what occurs when a male is approaching to the breeding season. Expression change in different trends led to a significant intersexual difference only in the breeding season.

## Discussion

### Males might increase diagnostic ability in green color

Our study offers the first evidence of sex-associated fluctuation in opsin expression for terrestrial vertebrates. The results of this study show that there are strong associations among seasonality, sexual maturity, hormone concentration, nuptial color, female choice, and opsin gene expression. Male lizards exhibited a significant increase in *rh2* and decrease in *lws* expression during maturation process, and a similar trend for mature males from nonbreeding to breeding season (Fig. 4A). We also found that this pattern could be replicated through artificial manipulation of testosterone, which resulted in even higher *rh2* and even lower *lws* expression. In contrast, the opposite pattern was observed in females between nonbreeding and breeding seasons (Fig. 4B). Statistically significant difference between sexes appeared only in the breeding season (Fig. 5), indicating that the diverse visual strategy between males and females might be critical in courtship behavior.

Modification in *lws* and *rh2* opsin expression might influence the chromatic or brightness sensitivity. Comparing to amino acid sequences of closely related lizards reported by previous literatures^24,48–51^, the four classes of cone opsins (LWS, Rh2, SWS2 and SWS1) in *T. viridipunctatus* (Supporting Information) were suspected to have maximum absorbance wavelength (λ_max_) around 560, 495, 435, and 365 nm, respectively. Without considering the effects of the oil droplets in cone cells, LWS opsin is the one with λ_max_ value that is closest to the nuptial color of the males (highest peak between 561 and 568 nm; Fig. 1). However, when the filtering effect of oil droplets was taken into consideration, the maximum sensitivity of Rh2 may lead to a red shift from 490 to 550 nm, which has been reported from other birds and lizards^24,51,52^. After the red shift, Rh2 best fits the male nuptial color. Therefore, elevation of *rh2* expression in males could be interpreted as a raise of sensitivity towards the green color. In addition to sensitivity, the increase of ‘evenness’ (the higher LWS decreased; and the lower Rh2 increased) between the two major classes of cones also improve the color discrimination ability.

However, these results raise the question of why male lizards raise their sensitivity and discrimination ability? The evidence for female choice in lizards is generally lacking^4,6,53,54^, but social systems are complex in lizards, and much of the sexual selection research has been conducted on a relatively small number of species. Whether female choice exists within *T. viridipunctatus* is an open question, but the findings here suggest that female choice might be occurring at some level, although more field and lab data is needed to fully test this hypothesis. We hypothesize that the courtship system of *T. viridipunctatus* could involve mate choice through both males and females^55^. Whereas many female lizards don’t display sexually dimorphic coloration, the green stripe on the ventrolateral side of females (see Fig. 1C) requires more study in this regard. It is possible that this female stripe might act as a signal to demonstrate whether a female is ‘ready to mate’ in the breeding season. The presence of this stripe may encourage male lizards to invest more mating efforts on a female that is in the best breeding condition. Advanced experiments, designed to test male choice for greenish females, will be our next goal to clarify the function of this stripe.

### Females might increase evaluation ability in brightness

In contrast to the findings discussed above, higher *lws* and lower *rh2* expression, compared to nonbreeding season, was exhibited in females during the breeding season (Fig. 4). As predicted from the previous experiments, males with greater T provision presented significantly higher coverages of green color (Fig. 2), and were significantly favored by the females (Fig. 3). It is noteworthy that testosterone did not change the color spectrum of the green spots, but increase the coverage area. Future studies of the corresponding change of gene expression in females might focus on the diagnostic ability in brightness and green area size which could reflect male quality, instead of focusing on the spectrum which does not change. In other words, the females might need the ability to effectively evaluate the size and coverage of nuptial coloration.

In most terrestrial vertebrates, two types of cones, single cones and double cones, contain opsin pigments. The former serves a function for chromatic vision, whereas the latter regulate achromatic function^56^. In the lizard retina, double cones containing LWS pigments have the highest abundance (40–50%)^49,50,56^. Therefore, the increased LWS pigments in female grass lizards might be largely expressed in the double cones and facilitate the sensitivity of females for brightness^50^. For males, enhancement of sensitivity to green color might be advantageous for allowing them to distinguish a reproductively mature female when her ventrolateral stripe turns green. For females, sensitivity to coverage and brightness could assist in enabling them to evaluate male quality.

It is also interesting to note trends with the two last opsin proteins (SWS1 and SWS2), including their synchronous rise in males in the breeding season (Fig. 4A), and also the synchronous reduction in females (Fig. 4B). Because lizards did not show UV reflection in their skin (Fig. 1), this alteration is unlikely to be relevant to their nuptial coloration. Indeed, alteration in SWS1 and SWS2 are seldom discussed within terrestrial vertebrates. One hypothesis predicted that the over expression of SWS1 in juvenile fish might provide a better prey-capture efficiency (Carleton, 2009)^57^, but in our own case, the reasons remain less obvious, and require further research. Currently, energy allocation might be the most likely explanation, which predicts that the raise of several opsins may lead to tradeoff of the others.

### Nuptial color as an honest signal

Although male ornaments and female preferences are a popular topic in evolutionary biology, the available literature specifically addressing direct female choice is comparatively rare in reptiles^25,54,58–61^. Our study raises the question of why female lizards might prefer specific colors in males. The handicap model suggests that the evolution of female preference associates with honest signals in males, which is presumably correlated with male quality and concomitant costs^6,62,63^. *Takydromus* lizards are usually non-territorial, inhabiting early-stage grasslands^46,64^. In this kind of environment, this species exhibits high population densities (in a long-term census program conducted in northern Taiwan^46^, more than 10,000 individuals have been marked within 6 years from a 500-meter transect). With such a high population density, a choosy female in the population could benefit from an effective mechanism for evaluating potential mates. With this kind of dense population, nuptial color could be a good indicator of male quality, reflecting their fighting success^65,66^, body condition^66,67^, health^67^, and other morphological traits associated with fitness^68^. In *T. viridipunctatus*, positive associations have also been found among green color, testosterone concentration, and locomotor performance in the wild (Lin *et al*., unpublished data). These ongoing studies may provide more information on the mating system of this species.

In summary, the coloration system of *T. viridipunctatus* might represent an unusual case of sexual selection in lizards, which offers some explanation for why both females and males display color signals. Evolution of a stable nuptial trait depends not only on the honesty of the signal, but also on the ability to precisely evaluate the traits. Our findings suggest that both sexes of the grass lizard regulate their opsin expression seasonally, which may have a strong influence on the detection or evaluation ability of nuptial coloration. This seasonal regulation might play an important role in the evolution of nuptial coloration.

## Materials and Methods

### Measurement of nuptial coloration

In order to quantify the nuptial color represented by the lizards during the breeding season, we measured spectral reflectance of six sexually mature males and females each in the breeding season, July of 2017. For each lizard, we measured three replicates, and then took the mean and standard deviation for (a) anterior green spots (on males) or brown belt (on females); (b) posterior green spots or brown belt; and (c) ventrolateral green line in both sexes. The detailed protocol of measurements is provided in Supporting Information.

### Testosterone treatments and husbandry of males

During the breeding season of May 2012, 60 male and 64 female *Takydromus viridipunctatus* were captured from Xindian, New Taipei City. Lizards were housed individually in terraria (30 × 20 × 2 cm, width × length × height) with L12: D12 circadian rhythm using artificial lighting with UVB lamps. Tile shelter and water was provided in each terrarium, with crickets regularly offered as food. All the collection, husbandry and treatment procedures followed Wildlife Conservation Act of Taiwan; where animal use protocols were approved by National Taiwan Normal University (license No. 101024).

In order to evaluate the change of lateral nuptial color from testosterone, male lizards were assigned randomly to three groups with different testosterone dosages: medium testosterone (medium T) treatment, high testosterone (high T) treatment, and control. Testosterone was obtained from SIGMA-ALDRICH (Code: 86500) and was dissolved in sesame oil into two concentrations: 1.25 mg/ml solution for medium T group, and 2.50 mg/ml solution for high T group. Lizards were offered testosterone every second day via pipetting a 2.0 μl solution on the dorsal surface for skin absorption (modified from similar experiment on lacertids)^69^; and the same amount of sesame oil was provided for the control group.

In order to confirm the validity of the hormone treatment, we measured testosterone level in the feces of each individual with enzyme immunoassay follow the protocol developed by Yeh *et al*.^70^ and Jiang *et al*.^71^. A Kruskal-Wallis test was used to test the difference in testosterone level in feces and enhancement of coloration among treatments. These values were also compared to a group of wild-caught lizards; among which the highest value we recorded in the wild was 173.22 ng/g.

### Quality and coverage change of nuptial color

The lateral side of each male lizard, before and after treatments, was photographed using a Nikon D7000 digital camera (Nikon D7000, Micro-Nikkor 60 mm f/2.8D lens) under the same posture with a SpyderCHECKR^TM^ reference color chart, and the area between fore and hind limbs was cut into a rectangular image. In order to quantify the nuptial greenish coloration, we measured the composition of RGB of a lizard by taking the average from five greenish spots chosen from the middle of lateral body using Adobe Photoshop CC. Differences before and after treatments, and among different testosterone groups, were compared by using repeated measures ANOVA. In order to quantify the coverage of nuptial spots, the greenish courtship coloration was selected using Adobe Photoshop CC, and the selected versus non-selected zones were transferred to a pure black-and-white image. The coverage ratio of the greenish area versus total lateral area was thus calculated by using Image-Pro Plus 5.1. The differences of the coverage ratio before and after manipulation were analyzed using Kruskal-Wallis tests to know the casual effect of T manipulation on the coverage lateral coloration.

### Female choice experiments

We designed an experimental terrarium for female choice (Fig. S1), which is similar to prior studies^72^ with detailed specification provided in Supporting Information. In order to test female preference on male coloration, we performed two series of pairings: medium T versus control, and high T versus control, which represent males with different green coverages. In each trial, two males from different treatments with similar SVL (less than 1 mm difference, <2.3%) were randomly moved into the two candidates’ chambers, and a female chooser was moved into the chooser’s chamber. The three lizards were settled in the chambers for a 10 min acclimation duration before the 1-hour record started. The response of the female was recorded by using a JVA GZ-E10 digital camera. The chambers for housing the lizards were cleaned by wiping 70% alcohol on a clean cloth on the surfaces before the next trial started. All experiments were conducted during sunny mornings, so that the candidates could be exposed to natural lights during the experiments. Paired t-tests were used to examine if females prefer to spend more time nearby one of the two males in the trials, which was defined as when the female was positioned in the choice zone and chosen by one of the candidates.

In order to clarify the function of lateral coloration as a diagnostic signal, we conducted a second experiment. Pairs of randomly chosen males, with SVL differences less than 1 mm (<2.3%), were chosen from the same treatment group. We masked the lateral color of one randomly chosen individual by using light brown paint to imitate male coloration in the nonbreeding season, and painted the other individual by using water as a sham control. We then tested whether the females prefer masked (brown) or unmasked (green) males by paired t-test.

### cDNA preparation for quantitative PCR

We compared the expression levels of the four opsin genes from males, females, and juveniles in the breeding (June) and nonbreeding (February) seasons. A total of three juvenile males, nine females (five from the nonbreeding season, and four from the breeding season), and 25 males (six from the nonbreeding and 19 from the breeding season) were collected from Xindian, New Taipei City. The 19 males collected during the breeding season were further separated into two groups: nine with T treatment (2.5 mg/ml testosterone solution), and 10 served as control (solvent oil provided only). The entire procedure of T treatment followed the description in the previous paragraph, with measurements, photos from the lateral side, and feces samples before and after treatments.

Lizards were euthanized by injecting lethal doses of benzocaine through their mouth, following the standard protocol approved by National Taiwan Normal University (license No. 101024). In order to minimize the time variation in opsin expression within a day, we standardize the euthanization between 21:00–24:00. Total RNA was extracted from dissected eyeballs drenched in RNAlater® Solutions using a QIAGEN® RNeasy Mini Kit, according to manufacturer’s protocol. RNA products had been measured concentration and examined quality in terms of absorption by Thermo NanoDrop 2000 spectophotometer. Before cDNA synthesis, DNase treatment to eliminate genomic DNA contamination was conducted. Reversed transcription of retinal RNA were executed via SuperScript® III First-Strand Synthesis SuperMix Kit (invitrogen).

### Primer design and quantitative PCR

High specificity to single species is required for primers used in qPCR. Therefore, full-length cDNA sequences of *T. viridipunctatus* from each class of opsin genes were obtained by using RACE (Tables S1 and S2; also see Supporting Information for detail). We designed four pairs of gene-specific primers by using Primer Express^®^ 3.0 (Applied Biosystems) software based on these full-length opsin sequences (Table S3). These primers were designed to amplify a 150-bp fragment and located cross two exon regions so that genomic DNA contamination could be detected by the size difference. Mitochondrial cytochrome *b* was chosen as reference gene for relative expression^73,74^.

We used StepOne^TM^ Plus real-time PCR system (Applied Biosystems) for qPCR detecting SYBR^®^ Green I Dye fluorescence. Standard curve and melting curve analysis were pre-conducted via StepOne Software v2.3 to qualify amplification efficiency and specificity in each pair of cone opsin primers, respectively (Figs S2 and S3). The concentration and detailed protocol for qPCR was provided in Supporting Information.

### Relative expression and cone opsin genes proportion analysis

Ct values of the five genes from every individual were exported to Microsoft Office Excel via StepOne Software v2.3. We calculated the relative gene expression with this formula:

$${{\rm{T}}}_{{\rm{i}}}/{{\rm{T}}}_{{\rm{ref}}}=(1/{(1+{{\rm{E}}}_{{\rm{i}}})}^{{{\rm{Ct}}}_{{\rm{i}}}})/(1/{(1+{{\rm{E}}}_{{\rm{ref}}})}^{{{\rm{Ct}}}_{{\rm{ref}}}})$$

where T_i_ and T_ref_ are target and reference gene expression respectively, and the products are multiples of expression. E_i_ and E_ref_ are amplification efficiency of each genes, which were calculated from standard curve analysis. We further identified the dominance genes and determined as a proportion for each gene normalized by the total cone opsin gene expressed, according to:

$${{\rm{T}}}_{{\rm{i}}}/{{\rm{T}}}_{{\rm{total}}}=(1/{(1+{{\rm{E}}}_{{\rm{i}}})}^{{{\rm{C}}}_{{\rm{ti}}}})/\sum (1/{(1+{{\rm{E}}}_{{\rm{i}}})}^{{{\rm{Ct}}}_{{\rm{i}}}})$$

Shapiro Wilk test and Brown-Forsythe test were used to check the assumptions of normality and equal variance. We then used t-test to examine the difference of gene expression between comparative groups, with all detailed statistics also provided in Supporting Information.

### Ethical approval

All the collection, husbandry and treatment procedures followed Wildlife Conservation Act of Taiwan; where animal use protocols were approved by National Taiwan Normal University (license No. 101024).

## Electronic supplementary material

Supplementary Information

## Data Availability

Opsin gene sequences are available on GenBank, and other relevant information is also available from the Dryad Digital Repository.
