# Supplementary material for: Opsin gene expression regulated by testosterone level in a sexually dimorphic lizard
Source: Sci Rep. 2018 Oct 30;8:16055. doi: 10.1038/s41598-018-34284-z (PMC6207759; doi:10.1038/s41598-018-34284-z)
Supplement: Supplementary file 1 — Supplementary Information [file 41598_2018_34284_MOESM1_ESM.docx]

**Supplementary Information**

**Opsin gene expression regulated by testosterone level in a sexually dimorphic lizard**

Wen-Hsuan TSENG, Jhan-Wei LIN, Chen-Han LOU, Ko-Huan LEE, Leang-Shin WU,

Tzi-Yuan WANG, Feng-Yu WANG*, Duncan J. IRSCHICK, Si-Min LIN*

**Measurement of nuptial coloration**

In order to quantify the nuptial color represented by the lizards during the breeding season, we measured spectral reflectance of six sexually mature males and females each in the breeding season, July of 2017**.** Reflectance spectra were measured by using a FLAME-S-XR1-ES spectrometer from Ocean Optics. An optical fiber probe connected to the detector approached the target sites or spots with a constant distance (~3 mm) to avoid variance in the numbers of photons captured. During the measurements, the fluorescent lights were turned off, and sunlight was blocked by a curtain. The only light source was a Mikropack DH-2000-BAL UV-VIS-NIR light source. One Teflon-coiled disc acted as calibration for 100% reflectance before every measurement. For each lizard, we measured three replicates, and then took the mean and standard deviation for (a) anterior green spots (on males) or brown belt (on females); (b) posterior green spots or brown belt; and (c) ventrolateral green line in both sexes.

**Testosterone concentration of males**

We measured testosterone level in the feces of each individual for confirming the validity of the hormone treatment. Testosterone was extracted with methanol by following the below procedure: 0.5 g feces mixing with 0.05 ml ddH2O and 0.4 ml methanol with 30 minutes of shake for mixing, follow with adding 0.3 ml petroleum ether and 15 seconds of shake, mixing suspension rotating (4℃, 3000 rpm) in centrifuge for 15 minutes. Testosterone in the ethanol natant liquid of the rotated suspension was measured with enzyme immunoassay described previously (Yeh et al., 2008, Jiang et al., 2009). In the enzyme immunoassay, fifty microliters of diluted serum and 50 μl of horseradish peroxidase-coupled testosterone (T) were added to a 96-well microtiter plate (Costar 3590) coated with anti-T monoclonal antibody T-1. After incubation at room temperature for 25 min and two washes with phosphate-buffered saline (PBS), the color was developed using 200 μl of 2.2 mM o-phenylenediamine in 0.003% H_2_O_2_ at room temperature for 30 min, then the reaction was stopped by addition of 50 μl of 8 N sulfuric acid and the optical density measured at 490 nm and compared to a T standard curve. The assay sensitivity was 15.6 pg/ml of T. The intra- and interassay coefficients of variation were 5-10% and 10-14%, respectively.

A Kruskal-Wallis test was used to test the difference in testosterone level in feces and enhancement of coloration among treatments. The test of testosterone level was to confirm the validness of hormone manipulation that males received testosterone treatment should have high testosterone level in their feces. These values were also compared to a group of wild-caught lizards; among which the highest value we recorded in the wild was 173.22 ng/g.

**Coverage change of nuptial color**

The lateral side of each male lizard, before and after treatments, was photographed by using a Nikon D7000 digital camera (Nikon D7000, Micro-Nikkor 60mm f/2.8D lens) under the same posture with a SpyderCHECKRTM reference color chart, and the area between fore and hind limbs was cut into a rectangular image. In order to quantify the nuptial greenish coloration, we measured the composition of RGB of a lizard by taking the average from five randomly chosen greenish spots using Adobe Photoshop CC. Differences before and after treatments, and among different testosterone groups, were compared by using repeated measures ANOVA. In order to quantify the coverage of nuptial spots, the greenish courtship coloration was selected using Adobe Photoshop CC, and the selected versus non-selected zones were transferred to a pure black-and-white image. The coverage ratio of the greenish area versus total lateral area was thus calculated by using Image-Pro Plus 5.1. Several cases of alteration in green nuptial color coverage were listed as followed in Figure S1.

(A) Low T treatment – case 1

**Figure S1.** Cases of green color coverage alteration before and after testosterone (T) treatments. (A) and (B) are cases from low T treatments; (C) and (D) are cases from high T treatments.

(B) Low T treatment – case 2

(C) High T treatment – case 1

(D) High T treatment – case 2

**Figure 2.** Continued.

**Female choice experiments**

To examine female preference, we designed an experimental terrarium (40$\times$20$\times$20 cm, width$\times$depth$\times$height; Figure S2) similar to prior studies (e.g., Hamilton & Sullivan, 2005). This terrarium was made of a 2 mm transparent acrylic sheet with no upper cover, which transmits > 92% of visible light (400 – 700 nm) and UV-A (315 – 400 nm), and > 73% UV-B (280 – 315 nm) regions. The terrarium was partitioned in two (40$\times$10 cm, width$\times$length) by a transparent acrylic sheet, and the partitioned space was defined as chooser’s chamber. The rest space (40$\times$10 cm, width$\times$length) was further separated by a nontransparent sheet into two candidates’ chambers (20$\times$10 cm, width$\times$length). Therefore, the two candidates were visually separated, and the chooser could watch through the transparent sheet and freely move to approach one of them. A string was attached on the top of the chamber, separating the chooser’s chamber into a 50% choice zone close to the candidates, and 50% nonchoice zone away from the candidates. This line helped to indicate the choosers’ relative position; the choosers were recorded to choose a candidate only when she was positioned in the choice zone.

In order to test female preference on male coloration, we performed two series of pairings: medium T versus control, and high T versus control, which represent males with different green coverages. In each trial, two males from different treatments with similar SVL (less than 1 mm difference, < 2.3%) were randomly moved into the two candidates’ chambers, and a female chooser was moved into the chooser’s chamber. The three lizards were settled in the chambers for a 10 min acclimation duration before the 1-hour record started. The response of the female was recorded by using a JVA GZ-E10 digital camera. The chambers for housing the lizards were cleaned by wiping 70% alcohol on a clean cloth on the surfaces before the next trial started. All experiments were conducted during sunny mornings, so that the candidates could be exposed to natural lights during the experiments. Paired t-tests were used to examine if females prefer to spend more time nearby one of the two males in the trials, which was defined as when the female was positioned in the choice zone and chosen by one of the candidates.

In order to clarify the function of lateral coloration as a diagnostic signal, we conducted a second experiment. Pairs of randomly chosen males, with SVL differences less than 1mm (< 2.3%), were chosen from the same treatment group. We masked the lateral color of one randomly chosen individual by using light brown paint to imitate male coloration in the nonbreeding season, and painted the other individual by using water as a sham control. We then tested whether the females prefer masked (brown) or unmasked (green) males by paired t-test.

**Figure S2.** Facility for female choice experiments.

**Cloning and sequencing of opsin genes from *Takydromus viridipunctatus***

***Sample collection***

Three *Takydromus viridipunctatus* was captured during their breeding seasons in July 2014. Lizards were euthanized by injecting lethal dose of benzocaine through their mouth, following the standard protocol approved by National Taiwan Normal University (license No. 101024). Eyes were enucleated and stored in RNAlater^®^ Solutions, which can stabilize retinal RNA for RNA extraction. In the meanwhile, muscle tissues from the tails were taken for genomic DNA extraction.

***Amplification of opsin genes from genomic DNA***

Genomic DNA was extracted via EasyPure Genomic DNA spin kit (Bioman) according to manufacturer's protocol. A roughly 220-bp conserved fragment was amplified by a pair of degenerate primers (OpF: gcgaattcgcntcancncaraarganga; OpR: gcaagct-tacrtanatnaynggrttrta, Carleton *et al.* 2000) from different opsin genes, mainly composed of relatively conserved exon4 region among vertebrate taxa. DNA purification was followed after excising the exact size of electrophoresed gel using QIAEX II Gel Extraction Kit. Purified products were cloned through pGEM^®^-T Easy Vector Systems to divide into single opsin gene. LB agar plates with ampicillin were incubated in 37ºC for 16 hours, then we randomly chose 24 clones for sequencing. Cloning products were sequenced in both directions on an ABI 3730 automatic sequencer by Biokit Biotechnology Corp. (Miaoli, Taiwan).

***Design of gene-specific primers***

Sequence analysis was conducted by SeqMan software from DNASTAR® Lasergene. We compared the sequences of the same assembled contigs to subject sequences on GenBank via BLAST in order to identify specific cone opsin gene. After comparison, exon 4 sequences of four classes of cone opsin genes were obtained from genomic DNA. We then designed four pairs of primers which were able to amplify all these four opsin genes (Table S1).

***RNA extraction and cDNA synthesis***

Total RNA was extracted from dissected eyeballs drenched in RNAlater^®^ Solutions using a QIAGEN^®^ RNeasy Mini Kit, according to manufacturer's protocol. RNA products had been measured concentration and examined quality in terms of absorption by Thermo NanoDrop 2000 spectophotometer. 5' and 3'-ready complementary DNA was synthesized through SMARTer™ RACE cDNA Amplification Kit for RACE amplification.

***RACE and cone opsins full-length sequencing***

RACE is a molecular method to obtain the full-length transcripts from overlapping one known fragments of a gene with its two opposite directional amplification sequences. 5'-RACE products were amplified by universal primers offered in the kit and reversed gene-specific primers designed from exon4 of genomic cone opsin genes, while 3'-RACE products were amplified by universal primers and forward gene-specific primers. DNA was purified after slicing the exact size of electrophoresed gel using QIAEX II Gel Extraction Kit. Purified 5' and 3' products were cloned through pGEM^®^-T Easy Vector Systems. LB agar plates with ampicillin were incubated in 37ºC for 16 hours, then we randomly chose 8 clones for sequencing. Sequences alignments of 5' and 3' products of each cone opsin could acquire full-length transcripts and obtain complete CDS by screening open reading frames (ORFs).

***Full length of cone opsin genes in Takydromus viridipunctatus***

*Takydromus viridipunctatus* express four classes of cone opsins that are sensitive to UV light (SWS1), blue (SWS2), green (Rh2) and red (LWS) wavelengths, which is in concordance with previous studies of most diurnal lizards. Full length cone opsin genes of *sws1* (1044 bp), *rh2* (1092 bp), and *lws* (1098 bp) were successfully cloned and sequenced from the total retina RNA; while *sws2* was successfully cloned for exon 1 – exon 4 (892 bp) (Table S2).

**Table S1.** Cone opsin gene-specific primer used in RACE amplification.

| Target |  | Sequence (5’→ 3’) | Size (bp) | Tm (ºC) |
| --- | --- | --- | --- | --- |
| *sws1* | F | GTGGTGGTGATGGTRGGCTCRTTCTGCC | 28 | 65.8 |
|  | R | GACGCAGGCGGACTTGGAGAAGAAGGCG | 28 | 67.2 |
| *sws2* | F | GTGACCAAGATGGTARTGGTGATGGTKATGGGC | 33 | 65.6 |
|  | R | GGAGGCCTTGRAGAAGACAGAGGGGATGG | 29 | 66.5 |
| *rh2* | F | GTTACYCGCATGGTGATCTTGATGGTGCTAGGG | 33 | 66.3 |
|  | R | GAGGGAYGAGCTCTTTGAGAAGAAGGCAGGCAC | 33 | 67.5 |
| *lws* | F | GAGGGAAGTATCAAGGATGGTCGTGGTTATGATC | 34 | 64.4 |
|  | R | ATGGTGGCGCTCTTTGCAAAGAAGGCAG | 28 | 62.8 |

**Table S2.** Complete cone opsin gene sequences of *Takydromus viridipunctatus.*

| Gene | Sequences |
| --- | --- |
| *sws1*  complete CDS | ATGTCCGGCGAGGAGGACTTCTACCTGTTTGAGAACATCTCCAAGGTGGGTCCCTGGGATGGCCCCCAGTACCACATCGCCCCAATGTGGGCCTTCTACTTCCAGACGGCCTTCATGGGCTTCGTCTTCTTCGCCGGGACGCCCCTCAACACCATCATCCTGGTGGTCACCGTCAAGTACAAGAAACTGCGCCAGCCGCTGAACTACATCCTAGTCAACATCTCCTTCGCCGGSTTCCTGTTCTGTGTCTTCTCCGTCTTCACCGTCTTCCTGGCCAGCTCGCAAGGCTACTTCTTCTTCGGGAGGCACATCTGCGCCCTGGAGGCCTTCCTGGGCTCGGTGGCAGGGCTGGTCACCGGCTGGTCCTTGGCCTTCCTCGCCTTCGAACGCTATATCGTCATCTGTAAGCCMTTTGGGAACTTCCGCTTCAACTCCAAGCACGCCCTTCTRGTGGTGGCMGCYACKTGGTTCATTGGAATCGGAGTCTCCATCCCACCCTTTTTTGGGTGGAGCAGGTTTATCCCCGAAGGCCTGCAGTGCTCCTGCGGCCCCGACTGgTACACGGTTGGCACCAAATACAAGAGCGAATACTATACCTGGTTCCTCTTCATCTTCTGCTTCATCGTGCCACTTACCCTCATCGTCTTCTCCTACTCGCAGCTCCTGGGCGCCCTCCGTGCCGTAGCAGCTCAGCAGCAGGAGTCGGCCACAACCCAGAAGGCCGAGCGGGAGGTCTCTCGTATGGTGGTGGTGATGGTGGGCTCGTTCTGCCTCTGCTACGTCCCATACGCCGCGCTTGCCATGTACATGGTGAACAACCGGGACCACGGCCTGGACCTGCGACTGGTCACCATCCCCGCCTTCTTCTCCAAGTCCGCCTGCGTCTACAACCCCATCATTTATTGCTTCATGAACAAGCAGTTTCGGGCTTGCATCATGGAGACCGTCTGTGGCAAACCCATGACCGATGGGTCTGATGTGTCCAGCTCAGCCCAGAAGACAGAGGTGTCCTCGGTTTCATCCAGCCAAGTCAGCCCCAGCTAA |
| *sws2*  exon 1 – 4 | ATGCACAGAGCCCGATCCGACTCCCAGGATGACATCCCGGAAGATTTCTTCATCCCAGTGCCCTTGGACGTTGCCAACATCACGACTCTAAGTCCTTTTCTGGTACCTCAGACTCACCTTGGTAGTCCAGGCCTCTTTATGAGCATGGCTGCCTTCATGTTCCTCTTGGTTGTCCTTGGGGTACCCATCAACGTCCTCACCATCTTCTGCACTTTCAAGTACAAGAAGCTCCGCTCCCACCTCAATTATATCTTGGTCAACCTTGCTGTGTCAAACCTGGTGGTTGTCTGCATTGGATCTACCACTGCCTTCTACAGCTTCTCCAAGATGTACTTCGTCATGGGAACTCTTGCTTGCAAGATAGAGGGCTTTGCTGCCACATTGGGGGGCATGGTGAGCCTATGGTCTCTGGCAGTTGTGGCCTTCGAACGATACTTGGTCATTTGCAAGCCTCTGGGAAACTTCACCTTCAGGGGCACCCATGCCATTGTTGGCTGTATCATCACATGGATCTTTGGCTTGGTGGCTTCAGTCCCACCCCTGTTTGGCTGGAGCAGGTATATTCCTGAGGGGCTGCAGTGCTCGTGTGGTCCTGATTGGTATACAACGAACAACAAGTGGAACAATGAGTCTTACGTCCTATTCCTCTTTTCCTTCTGCTTTGGAGTGCCCCTGTCTGTTATCGTCTTTTCTTATGGTCGTCTTCTGATAACCCTGCGGGCAGTTGCCAAGCAGCAGGAGCAGTCAGCCACCACACAGAAGGCTGAGAGAGAGGTGACCAAGATGGTAGTGGTGATGGTGATGGGCTTCCTGGTGTGCTGGCTGCCATACGCCTCCTTTGCATTGTGGGTAGTGACCCACCGGGGAGAACCTTTTGACGTTCGCCTGGCCT |
| *rh2*  complete CDS | ATGAATGGAACAGAAGGTATCAATTTTTATGTGCCTCTTTCCAACAAGACAGGGCTGGTGCGGAGCCCCTTTGAATATCCCCAGTATTACCTAGCGGAACCCTGGAAGTACAAAATGGTGTGCTGCTACATCTTCTTCCTCATCTCCACGGGGCTGCCCATCAACCTCCTCACCCTCCTTGTGACCTTCAAACATAAGAAGCTACGACAGCCGCTCAACTATATCCTGGTTAACCTGGCGGTAGCAGACCTCTTCATGGCCTGCTTTGGCTTCACGGTCACCTTCTACACAGCCTGGAATGGCTACTTCATATTTGGTCCCATCGGCTGTGCCATTGAGGGCTTCTTTGCAACACTGGGAGGTCAGGTTGCTCTCTGGTCTCTGGTTGTTCTAGCCATAGAGCGCTATATTGTGGTCTGTAAACCAATGGGAAACTTCCGCTTCTCTTCCTCTCATGCCTTGATGGGCATTGCTTTTACTTGGTTTATGTCCCTGTCCTGTGCATGTCCACCTCTTTTTGGTTGGTCCAGATATATACCTGAGGGGATGCAGTGTTCCTGTGGCCCAGACTACTACACCCTCAACCCTGATTATCACAATGAGTCCTACGTTGTCTATATGTTTCTCATCCACTTCGTTATTCCCGTGGTGGTCATCTTCTTTTCCTATGGGCGGCTCATATGCAAAGTCCGAGAGGCAGCTGCTCAGCAGCAAGAGTCTGCAAGCACCCAGAAGGCAGAGAAAGAAGTTACCCGCATGGTGATCTTGATGGTGCTAGGGTTTATGCTTGCCTGGACGCCCTATGCCGTGGTGGCATTCTGGATCTTCACCAACAAAGGGGCAGACTTTTCTGCTACGCTCATGTCAGTGCCTGCCTTCTTCTCAAAGAGCTCRTCCCTCTACAATCCCATCATCTATGTCCTCATGAACAAACAGTTCCGTAATTGCATGATCACCACAATCTGCTGTGGTAAAAACCCCTTTGGGGACGATGATGTCTCATCAACTGTATCCCAAAGCAAAACCGAGGTATCCTCTGTCTCCTCCAGCCAAGTGTCACCTGCATAG |
| *lws*  complete CDS | ATGACAGAAGCCTGGGATGTGGGTGTCTTTGCTGCCCGCCGGCGCAATGAAGATGACGACACCACCAGAGACAGCCTGTTTACTTACACCAACAGCAACAATACTCGGGACCCTTTTGAAGGTCCAAACTATCACATTGCACCACGATGGGTCTATAACATCACTTCTCTCTGGATGATCTTTGTGGTTGTTGCCTCAGTCTTCACTAATGGTTTGGTATTGGTGGCCACTGCCAAATTCAAGAAGCTACGGCATCCACTCAACTGGATTTTGGTGAACCTGGCAATAGCTGACCTGGGTGAGACAGTTATTGCCAGCACCATCAGCGTCATCAACCAGATCTTTGGCTATTTCATTCTTGGTCACCCCATGTGTGTGTTGGAAGGATACACCGTATCTGCTTGTGGCATAACAGCCCTCTGGTCTTTAGCCATTATCTCCTGGGAGCGTTGGGTTGTTGTCTGCAAGCCCTTTGGAAATGTCAAGTTTGATGCCAAATTGGCTATGGCTGGCATTCTCTTCTCCTGGATATGGTCTTGTATTTGGACAGCACCACCCATCTTTGGCTGGAGTAGGTACTGGCCCCATGGTCTGAAAACATCATGCGGTCCAGATGTATTCAGTGGCAGTGAAGATCCTGGAGTTCAGTCTTACATGGTTGTGCTCATGATCACCTGTTGCTTCCTCCCCCTGGCTGTCATCATTCTCTGCTACCTGCAAGTGTGGCTCGCTATCCGTGCGGTTGCTGCCCAGCAAAAGGAGTCAGAATCTACGCAGAAAGCTGAGAGGGAAGTATCAAGGATGGTCGTGGTTATGATCATTGCCTATTGCTTCTGCTGGGGACCATATACGATCTTTGCCTGCTTTGCTGCTGCCAACCCAGGCTATGCCTTCCACCCCCTTGCAGCTGCCCTGCCTGCCTTCTTTGCAAAGAGCGCCACCATCTACAACCCCATTATCTACGTCTTCATGAACAGACAGTTCCGTAACTGCATATTGCAGCTTTTTGGCAAGAAGGTGGATGATGGCTCTGAGGTCTCTTCCACTTCCCGCACTGAAGTCTCCTCTGTCTCTAATTCTTCTGTATCGCCATCATAA |

**Protocol for quantitative PCR (qPCR)**

***Primer design***

High specificity to single species is required for primers used in qPCR. Therefore, full-length cDNA sequences of *T. viridipunctatus* from each class of opsin gene were obtained by using RACE (details see Appendix 1). We designed appropriate primers by using Primer Express^®^ 3.0 (Applied Biosystems) software (Table S3). Mitochondrial cytochrome *b* was chosen as reference gene for relative expression. Primers were designed to amplify a 150-bp fragment and located cross two exon regions so that genomic DNA contamination could be detected by the size difference.

***Quantitative PCR***

We used StepOne^TM^ Plus real-time PCR system (Applied Biosystems) for qPCR detecting SYBR^®^ Green I Dye fluorescence. Standard curve and melting curve analysis were pre-conducted via StepOne Software v2.3 to qualify amplification efficiency and specificity in each pair of cone opsin primers. In standard curve construction, 4 serial 5X diluted standard retinal cDNA (50, 10, 2, 0.4 ng/µl) were all amplified to examine primer efficiency. Every 20-µl reaction composed of 2X SYBR™ Green Master Mix, 0.5 µl for one pair of 5M qPCR primers, and 10 ng retinal cDNA as template. Each reaction was performed using a ABI 7300 Real Time PCR System (Applied Biosystems, California, USA) with the following steps: 1 cycle of 50°C for 2 min and 95°C for 10 min, followed by 40 cycles of 95°C for 15 sec and 60°C for 40 sec, ended by 1 cycle of 95°C for 15 sec and 60°C for 1 min and 95°C for 15 sec for melting curve. For each gene and each individual, three replicates were conducted for the sake of reducing artificial manipulated-caused deviation.

The two axes in result plots were Ct values against RNA concentrations, for which all primers should attain efficiency values in a range between 90% and 110% (the slope of regression line would be -3.32 if efficiency equals to 100%), with correlation coefficient over 0.98 before proceeding to following experiments. Regression lines in standard curve analysis and melting curves for each primer are shown in Figures S2 and S3, respectively.

***Relative expression and cone opsin genes proportion analysis***

Ct values of the five genes from every individual were exported to Microsoft Office Excel via StepOne Software v2.3. We calculated the relative gene expression with this formula:

T_i_/T_ref_=(1/(1+E_i_)^Ct_i_)/(1/(1+E_ref_)^Ct_ref_)

Where T_i_ and T_ref_ are target and reference gene expression respectively, and the products are multiples of expression. E_i_ and E_ref_ are amplification efficiency of each genes, which were calculated from standard curve analysis. We further identified the dominance genes and determined as a proportion for each gene normalized by the total cone opsin gene expressed, according to:

T_i_/T_total_=(1/(1+E_i_)^Ct_i_)/ Σ(1/(1+E_i_)^Ct_i_)

We used t-test to examine the difference of gene expression between comparative groups. Shapiro Wilk test and Brown-Forsythe test were used to check the assumptions of normality and equal variance. In the comparisons of males, almost all opsin data fitted the assumptions of normality and homogeneity (all p>0.05) except the comparison of *sws1* and *lws* between males with T treatment and control that had significantly different variance (*sws1*: *F*_1,18_=24.88, *p*<0.0001; *lws*: *F*_1,18_=7.81, *p*=0.0120). We used t-test assuming unequal variance to test these comparisons. In order to exclude the possibility that variation from testosterone impact was generated by different body condition at first or caused by treatment, we used ANCOVA with weight before treatment and weight difference between before and after treatment as covariates in the comparisons between males with testosterone treatment and control. None of these weight parameters affect the opsin expression between treatments in any comparison (all *p*>0.05). In the comparisons of females, only the *sws1* data in breeding females was not normal distributed, we thus used Wilcoxon rank sum test to compared *sws1* expression between breeding and nonbreeding females. The rest comparisons in females were performed with t test. All data of comparisons between sexes fitted the assumptions of t test.

**Table S3.** Primers used for quantitative PCR (qPCR) in this study.

| Target | Sequences (5’ → 3’) | | Tm (℃) | Amplified efficiency (%) |
| --- | --- | --- | --- | --- |
| *lws* | F | GGTTGTGCTCATGATCACCTGTT | 60 | 105.9 |
|  | R | ACCATCCTTGATACTTCCCTCTCA | 59 |  |
| *rh2* | F | CGTGGTGGCATTCTGGATCT | 60 | 106.9 |
|  | R | GTGATCATGCAATTACGGAACTG | 58 |  |
| *sws1* | F | AAGTCCGCCTGCGTCTACAA | 59 | 98.6 |
|  | R | TGAAACCGAGGACACCTCTGT | 58 |  |
| *sws2* | F | CCATGCCATTGTTGGCTGTA | 59 | 101.6 |
|  | R | TCATTGTTCCACTTGTTGTTCGT | 58 |  |
| cytochrome *b* | F | GTTCCTTACATTGGGACAACACTTG | 60 | 109.2 |
|  | R | TTCATGCAGGAAAAGGAGATGA | 58 |  |

**A**


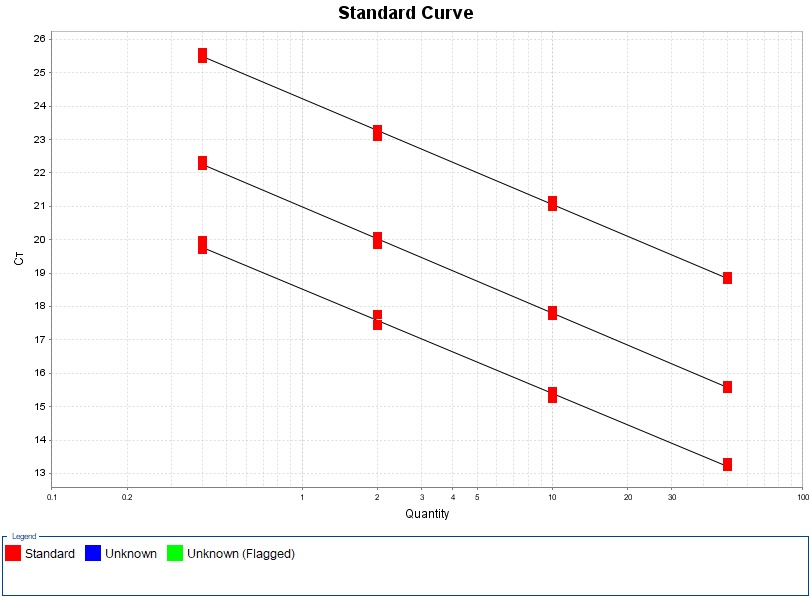


*cyt b*

*lws*

*rh2*

Quantity (ng/μl)

| Target: *rh2* | Slope: -3.166 | Y-Inter: 24.226 | R^2^: 0.999 | Eff%: 106.936 |
| --- | --- | --- | --- | --- |
| Target: *lws* | Slope: -3.187 | Y-Inter: 20.994 | R^2^: 0.999 | Eff%: 105.947 |
| Target: *cyt b* | Slope: -3.12 | Y-Inter: 18.525 | R^2^: 0.998 | Eff%: 109.196 |

**B**


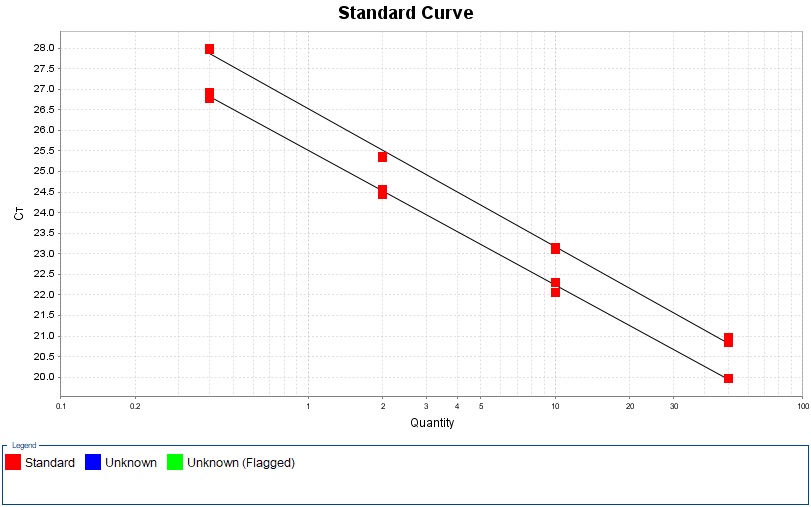


*sws1*

*sws2*

Quantity (ng/μl)

| Target: *sws1* | Slope: -3.355 | Y-Inter: 26.521 | R^2^: 0.998 | Eff%: 98.618 |
| --- | --- | --- | --- | --- |
| Target: *sws2* | Slope: -3.284 | Y-Inter: 25.514 | R^2^: 0.999 | Eff%: 101.616 |

**Figure S2.** Regression lines in standard curve analysis of qPCR primers plotted as Ct values against 4 diluted RNA concentrations for (A) cytochrome *b*, *lws*, *rh2*; and (B) *sws1* and *sws2* genes.

**A**


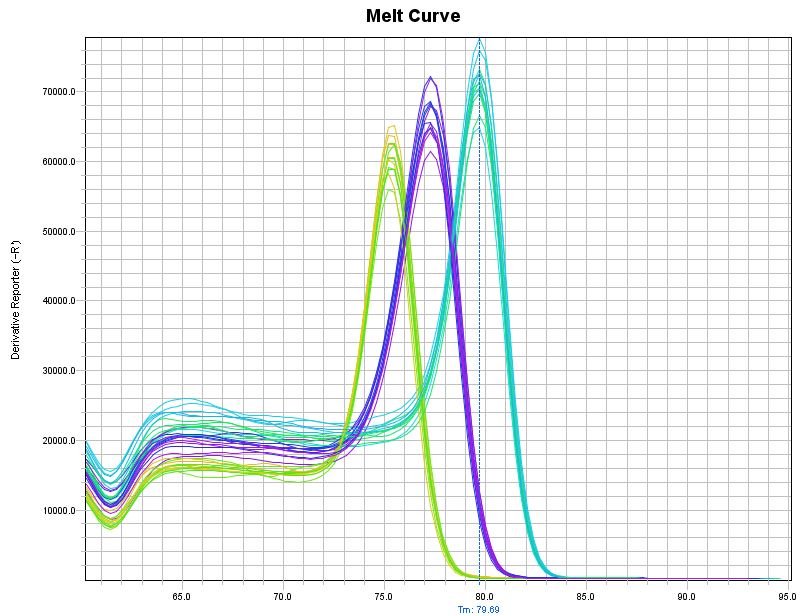


***cyt b***

***rh2***

***lws***

**B**

***sws2***


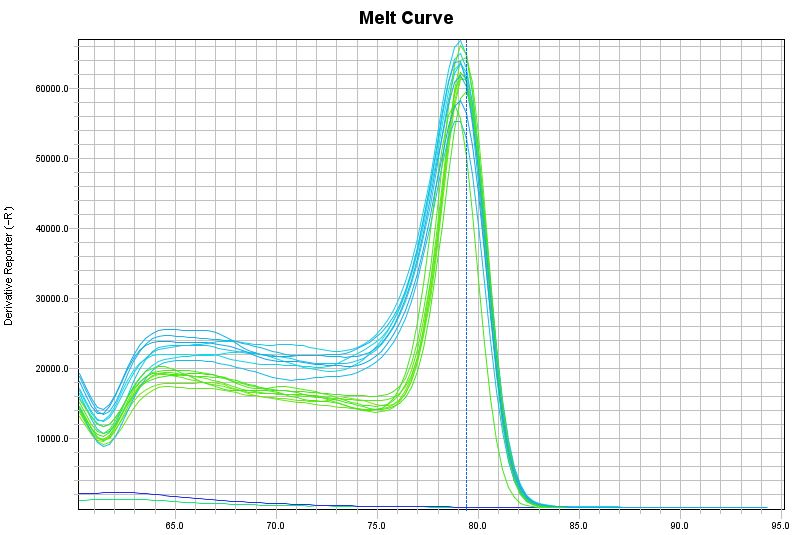


***sws1***

**Figure S3.** Melting curves for (A) cytochrome *b*, *lws*, *rh2*; and (B) *sws1* and *sws2* genes.
